# Pulmonary zygomycosis: A clinicopathological study

**DOI:** 10.4103/0970-2113.76297

**Published:** 2011

**Authors:** Sundaram Challa, Shantveer G. Uppin, Megha S. Uppin, Roshni T. Paul, Aruna K. Prayaga, Manmadha T. Rao

**Affiliations:** *Department of Pathology, Nizam’s Institute of Medical Sciences, Hyderabad, Andhra Pradesh, India*; 1 *Department of General Medicine, Nizam’s Institute of Medical Sciences, Hyderabad, Andhra Pradesh, India*

**Keywords:** Diabetes mellitus, lung, pathology, zygomycosis

## Abstract

**Background and Objective:**

Zygomycosis is an emerging infection worldwide. Pulmonary zygomycosis (PZ) is uncommon with only few reported series from India.

**Materials and Methods:**

All cases of PZ diagnosed on histopathology between 1995 and 2008 were included. Clinical and imageological findings were noted in all cases. Routine hematoxylin and eosin-stained sections were studied to assess the pathology; Gomori’s methenamine silver (GMS), periodic acid Schiff were done for delineating fungal morphology. Culture reports were collected wherever available. Treatment and outcome details were noted.

**Results:**

Seven patients were diagnosed with PZ during the study period, which included six males and one female patient. Six of these had diabetes mellitus (DM) and one patient was on chemotherapy for the treatment of Hodgkin’s lymphoma. Fever and cough were the most common presenting features. Consolidation with or without cavitation was seen in six patients and lung abscess with fungal ball in one patient. All six patients with DM had upper lobe involvement and four had multiple lesions. Histological sections revealed necrotizing inflammation, hemorrhagic infarcts and angioinvasion. Culture was available in two patients, which grew Rhizopus oryzae. Five patients succumbed to disease and remaining two were lost to follow-up.

**Conclusion:**

Diabetes mellitus is the most common predisposing factor for PZ and carries high mortality.

## INTRODUCTION

Zygomycosis is an emerging infection worldwide. Most of the infections occur in a setting of immunosuppressed state. Rhinocerebral form is the most common form.[1–7] Pulmonary zygomycosis (PZ) is uncommon and its incidence varies from 6% to 24% in various reported series.[3,4] The pulmonary manifestations are protean and include cavitory lesions, pneumonia, solitary nodule, or disseminated lesions.[1,2] Diagnosis is difficult to establish and untreated patients succumb to the disease. There are very few reported series of PZ from India.[8–16] In this paper, we report a series of seven patients of PZ diagnosed on histology and discuss the difficulties in establishing the diagnosis.

## MATERIALS AND METHODS

All the patients diagnosed as PZ on tissue sections either on biopsy/lobectomy/autopsy during the period 1995–2008 were included in the study. Morphology of fungal hyphae which were broad aseptate and branching irregularly were identified as zygomycetes. The demographic data, predisposing risk factors, clinical and radiological features, and bronchoscopic findings were noted from medical records. The culture reports of sputum and bronchial washings were collected whenever available. The hematoxylin and eosin slides were reviewed for the pathology and special stains including Gomori’s methenamine silver (GMS), periodic acid Schiff (PAS) were done for delineating fungal morphology. Treatment given, complications and outcome, were noted from records.

## RESULTS

There were seven patients diagnosed with PZ in the study period (14 years). There were six males and one female with age ranging from 40–60 years (mean 46.7 years). The demographic data, predisposing risk factors, clinical features, radiological features, pathology, culture, treatment, and outcome were given in Table 1.

**Table 1:** Pulmonary zygomycosis–age, gender, clinical, and imageological features, pathology treatment and outcome

Case no.	Age (years)/gender	Predisposing factors	Clinical features	Radiological/ bronchoscopic findings	Pathology and culture	Treatment and outcome
1	48/M	DM	Fever, cough, SOB – 15 days. No response to antibiotics Clinical Diagnosis: Rt. UL abscess	CT chest – Rt UL abscess with fungal ball	Bronchial biopsy showed necrotizing inflammation and fungal ball Culture: Sputum and bronchial wash negative for fungi	Amphoterecin B. Improved symptomatically but discharged against medical advice and lost to follow-up
2	45/M	Diabetic ketoacidosis	Fever with chills and rigors, hemoptysis Clinical Diagnosis – Rt side pneumonia	CT chest – Rt. Upper and midzone consolidation. Bronchoscopy – endobronchial necrotic lesion eroding the Rt. main bronchus medially with greenish slough	Bronchial biopsy showed necrotic lesion with fungal hyphae Culture: Bronchial washings grew Rhizopus oryzae	Amphoterecin B. Had a massive bout of hemoptysis and died.
3	45/F	DM	Fever, cough, SOB – 10 days, no response to antibiotics Clinical Diagnosis: Bilateral pneumonia	X-ray chest.– Rt UL, Lt LL consolidation, ARDS	Post mortem biopsy from Rt UL shows intraalveolar and interstitial fungal hyphae. Culture: Sputum negative for AFB and fungi	Patient succumbed to disease before diagnosis was established.
4	60/M	DM	SOB – 10 days. Bilateral pedal edema – 1 month. No response to antibiotics. Clinical Diagnosis: Malignancy	CT chest – Cavitating mass lesions, Rt. UL and Rt. LL Clinical Diagnosis: Malignancy	Post mortem biopsy Rt. apical lesion shows necrotizing lesion with intra cavitary fungi with angioinvasion.	Patient succumbed to disease before diagnosis was established.
5	40/M	Diabetic ketoacidosis	Fever, productive cough, foul swelling. Rt. Side chest pain – 4 months. Loss of appetite, weight. Clinical Diagnosis: Pneumonia No response to antibiotics	CECT chest – consolidation in right UL with loculated pleural collection. Bronchoscopy – Thick pus and blackish granulation tissue, obstructing the right bronchus.	Bronchial biopsy – Necrotizing inflammation with fungal hyphae and angioinvasion Culture: Sputum and bronchial washings negative for AFB, fungi	Clinical and radiological improvement with Amphoterecin B. Did not complete treatment and left against medical advice.
6	40/M	HL on CT	Fever, cough – 10 days. No response to antibiotics. Clinical diagnosis: pneumonia	X–ray chest – opacity Lt. lower zone lung. Rigid bronchoscopy – Bleeding from Lt. bronchus	Lt. lower lobectomy with part of rib and soft tissues excised. Infarction of lung with neutrophilic infiltrates fungal hyphae and angio invasion, invasion into bone, skeletal muscle, soft tissues present. Culture: Sputum was negative for AFB and fungi	Amphoterecin B after diagnosis, but succumbed to disease within one week
7	49/M	DM	Fever, cough, SOB, chest pain – 15 days. Streaks of hemoptysis Clinical diagnosis: pneumonia	X-ray chest – consolidation of lt. lingular lobe. CECT - Infiltrates in the Rt. LL, cavitating consolidation Lt. UL. Bronchoscopy – necrotizing pneumonia	Patient succumbed to a massive bout of hemoptysis and a complete autopsy was performed. At autopsy, there were bilateral hemorrhagic infarcts in the lung invaded by fungi and angioinvasion. Culture of sputum and bronchial washings and tissue submitted at autopsy grew Rhizopus oryzae.	Amphoterecin B following culture report of sputum. Patient succumbed to massive bout of hemoptysis and autopsy was performed.

Rt. – right; Lt. – left; UL – upper lobe; LL – lower lobe; CECT – contrast enhanced computed tomogram; AFB – acid fast bacilli; ARDS - adult respiratory distress syndrome; DM – diabetes mellitus, SOB: shortness of breath

### Predisposing risk factors

One was a patient of Hodgkin’s lymphoma on chemotherapy who developed pneumonia which did not respond to antibiotics. The remaining patients had diabetes mellitus (DM) with two of them in ketoacidosis. In two patients, DM was diagnosed at the present admission.

### Clinical presentation

Fever and cough were the most common presenting features (6/7). The other clinical features included shortness of breath, pedal edema, and chest pain. Two patients had massive hemopytsis and succumbed to it.

### Radiological features

**Figure 1:**
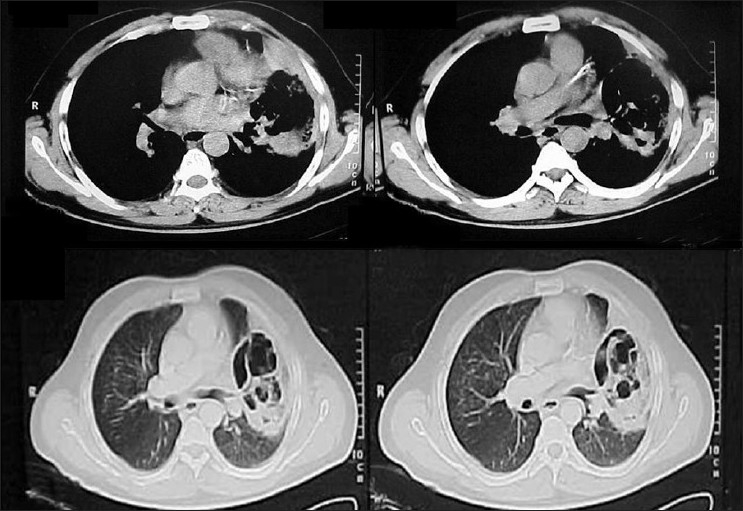
CT chest shows soft tissue attenuating lesion with multiple cavities involving left upper lobe and lingula

Consolidation with or without cavitation was seen in five patients on plain radiographs and CT chest [Figure 1]. Lung abscess with fungal ball was seen in one patient. Only plain radiographs were available in two patients and all the others had CT scans. In all six patients with DM, upper lobe involvement was seen and in four patients multiple lesions were seen.

### Bronchoscopic features

Reddish granular friable material obstructing the bronchus was seen in three patients. Thick pus and granulation tissue was seen in one patient. Endobronchial lesion eroding the main bronchus and covered with greenish slough was seen in one patient.

### Pathology

**Figure 2:**
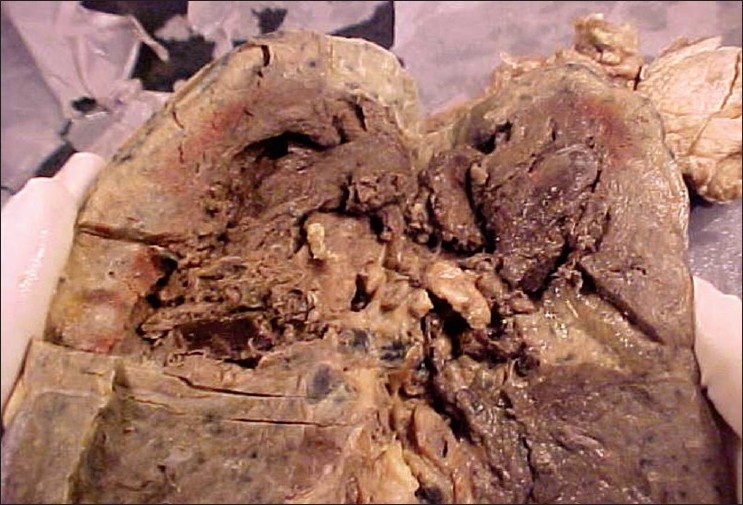
Autopsy specimen of lung showing necrotizing lesion with cavity in the upper lobe (black arrow)

The tissue submitted for histopathology included bronchial biopsy in three, postmortem biopsy in two, lobectomy specimen with overlying rib and soft tissues in one. Autopsy was done in one patient which showed cavitary lesion in the upper lobe [Figure 2]. The biopsies showed necrosis with neutrophilic infiltrate and invasion by fungal hyphae. Lobectomy specimen showed hemorrhagic infarcts and angio-invasion with extension, into bone, skeletal muscle, adipose tissue, and collagen. In the autopsy case, lungs showed bilateral hemorrhagic infarcts with angioinvasion and infiltration by fungal hyphae.

**Figure 3:**
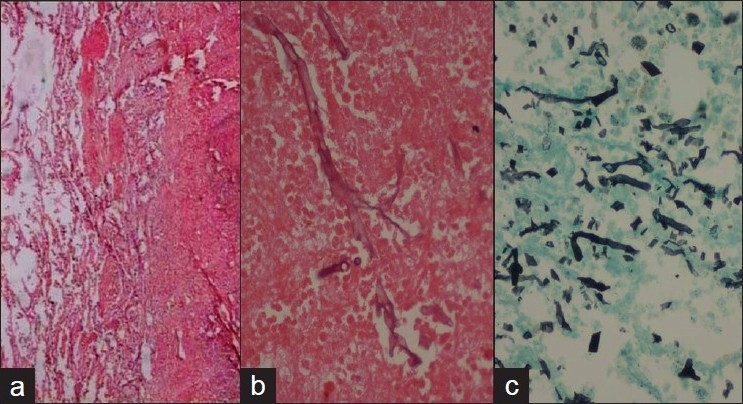
Histological sections showing (a) hemorrhagic infarct in lung (H and E; ×40), (b) broad aseptate fungal hyphae within the necrotic tissue (H and E; ×400), and (c) Gomori’s silver methenamine stain highlighting the fungal hyphae (GMS stain; ×200)

**Figure 4:**
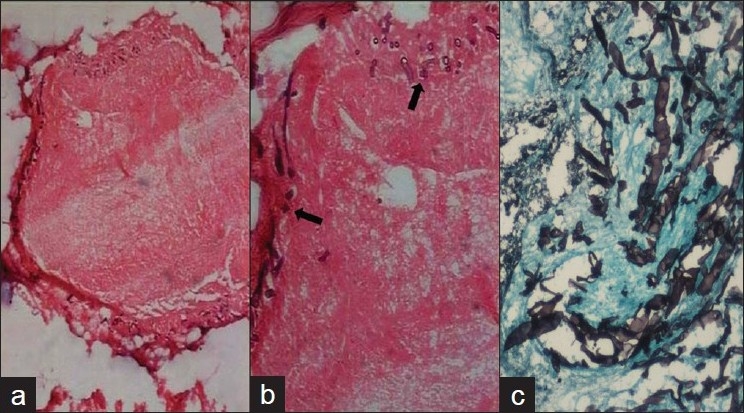
Histological sections showing (a) thrombosed vessel (H and E; ×40), (b) fungal hyphae (black arrows) invading the vessel wall (H and E; ×100), and (c) Gomori’s silver methenamine stain highlighting angioinvasion by aseptate broad hyphae of Zygomycetes species (GMS; ×200)

There was no other organ involvement by zygomycetes. The fungal hyphae in all samples were broad, aseptate with irregular or right angle branching. The hyphae were pale and hyaline on H and E and were better delineated on GMS and PAS [Figures 3 and 4].

### Culture

Cultures of sputum and bronchial washings were negative for acid fast bacilli and fungi in four samples and grew Rhizopus oryzae in two. Tissue culture was submitted only in autopsy case and grew Rhizopus oryzae.

### Treatment and outcome

Two patients succumbed to disease before a diagnosis was established. None of the patients received antituberculous treatment. The other five patients were started on Amphotericin B showed symptomatic improvement. Two of these did not complete the treatment and left against medical advice and two succumbed to massive bouts of hemoptysis. One patient succumbed to disease within 1 week of starting treatment, despite surgical debridement and Amphotericin B.

## DISCUSSION

Zygomycosis is an opportunistic infection. The common risk factors include DM with ketoacidosis and hematological neoplasms like lymphoma and leukemia on chemotherapy.[17] In our series DM was the most common risk factor (6/7 patients). The remaining one patient was on chemotherapy for Hodgkin lymphoma. In the reported series from the west, hematologic disease like lymphoma and leukemia on chemotherapy constitute the commonest predisposing factor for PZ.[2,3] Pulmonary zygomycosis is an emerging fungal infection among patients with cancer and especially presents as a breakthrough infection if treatment with antifungal agents effective against Aspergillus species are given.[18] However, in the series reported from India, uncontrolled DM was the commonest risk factor for most types of zygomycosis including pulmonary form.[5,19]

Fever and cough were the most common presenting features in our patients as reported in earlier series.[1,2] Other features reported include dyspnoea, malaise, chest pain, and hemoptysis.[1,2] Rare presentations include recurrent laryngeal nerve palsy.[12] None of the clinical features were specific or diagnostic for zygomycosis. Radiologically consolidation was the commonest finding followed by cavitory lesions and abscesses. All six patients with DM in our series had upper lobe involvement and four patients had multiple lesions. The radiological features reported on chest radiographs include infiltrates, nodular masses which may be solitary or diffuse.[2]

However, the sensitivity of chest radiographs is lower when compared to contrast enhanced CT in identifying lesions of pulmonary fungal infections.[20]

Bulky, nodular, and cavitary lesion with upper lobe predominance on CT favor a diagnosis of PZ.[21,22,23] Multiple nodules pleural effusion and associated sinus involvement also favor a diagnosis of PZ.[18,20] Though necrotizing and cavitary consolidation with preferential upper lobe involvement in all and multiple lesions in 4/6 diabetic patients were seen in our series, a clinical or radiological diagnosis was not made in any patient. Associated sinus involvement was not seen in our patients and pleural effusion was seen in one patient only.

Bronchial biopsy was the most common diagnostic modality in our series followed by sputum cytology. The diagnostic yield was better with bronchial biopsy as diagnosis could be established in three patients on bronchial biopsy whereas sputum cytology was positive in only one patient and negative in two patients. The diagnostic modalities for PZ include culture of sputum, bronchioloalveolar lavage or pleural fluid. However, the sensitivity of these modalities for diagnosis is very low. Definitive diagnosis requires histologic demonstration of tissue invasion by broad aseptate hyphae along with culture of tissue. Hence, percutaneous needle biopsy, open biopsy or bronchoscopic biopsy are advocated for definite diagnosis of PZ.[1,18] Diagnosis could be established antemortem in five of our patients.

The morphology of the fungi in tissue sections was characteristic and with the help of special stains, the diagnosis was made in all patients. However, culture positivity was present in only two patients. An increase in culture positivity of up to 70%, since 2000 has been reported.[3] Crushing of tissue during biopsy may decrease culture yield as the hyphae of zygomycetes are aseptate and liable to damage during tissue manipulation.[1] Rhizopus oryzae was the most commonly isolated organism as seen in both our patients. PZ with Cunnighamella species is an independent risk factor associated with worse prognosis when compared to Rhizopus species.[3]

Treatment for PZ includes Amphoterecin B and surgery and both are independently associated with a decreased risk of mortality.[3] Three of our patients were started on Amphoterecin B, one refused treatment and one patient who had extension of infection into chest wall had lobectomy and debridement. The cause of death was massive hemoptysis in two patients. Gupta *et al*., reported similar event.[8] There was no significant improvement reported in the outcome of PZ in the past few decades.[1] This is mainly due to delay in diagnosis and instituting treatment as seen in all our patients.

With the increasing number of diabetic and other immunosuppressed individuals, high index of suspicion on clinical and radiological features is essential to make a diagnosis of PZ. A diabetic patient presenting with fever and having upper lobe consolidation with or without cavitation, not responding to usual antibiotic treatment should arouse the suspicion of PZ. Early diagnosis and starting treatment immediately may change the outcome.
